# Asymptomatic school children and adults are important for the human infectious reservoir for *Plasmodium falciparum* malaria in an area of low endemicity in The Gambia

**DOI:** 10.1016/j.jinf.2025.106507

**Published:** 2025-07

**Authors:** Harouna M. Soumare, Sara Lynn Blanken, Abdullahi Ahmad, Michael Ooko, Pa Modou Gaye, Lamin Jadama, Muhammed M. Camara, Ebrima A. Jawara, Kjerstin Lanke, Amie Kolleh Njie, Michael Mendy, Blessed Etoketim, Lamin Camara, Mamadou O. Ndiath, Bakary Conteh, Nuredin Muhammed, Seyi Soremekun, Abdoullah Nyassi, Annette Erhart, Chris Drakeley, Teun Bousema, Umberto D'Alessandro, Marta Moreno

**Affiliations:** a Medical Research Council Unit The Gambia at the London School of Hygiene & Tropical Medicine, Banjul, Gambia; b Department of Medical Microbiology, Radboud University Medical Center, Nijmegen, The Netherlands; c Department of Infection Biology, London School of Hygiene & Tropical Medicine, Keppel Street, London, UK

**Keywords:** *Plasmodium falciparum*, Asymptomatic, Symptomatic, Anopheles, Transmission, Membrane feeding assay

## Abstract

**Objectives:**

In The Gambia, the scale-up of malaria control interventions in the past decades resulted in a substantial decrease of the malaria burden. However, low levels of malaria transmission persist.

**Methods:**

We conducted an observational cohort study in eastern Gambia to better understand the relative contribution of symptomatic and asymptomatic malaria infections to the infectious reservoir. Parasite and gametocyte carriage were determined by molecular methods. Infectiousness to mosquitoes was assessed by mosquito membrane feeding assays on a subset of symptomatic and asymptomatic individuals identified by passive case detection and community surveys.

**Results:**

Incidence of clinical malaria was 1.46 episodes/100 person-months. Prevalence of malaria infection as determined by PCR in community surveys was 10.5%. Among asymptomatic malaria-infected individuals, total parasite density was positively associated with gametocyte density (β = 0.40; P < .0001). Mosquito infection rates in membrane feeding experiments were positively associated with gametocyte density (β = 2.81; P < 0.0001). More than 84% of mosquito infections occurred in asymptomatic individuals with patent infections, with the highest contribution from older children (40.3%), and adolescents and adults (45.5%). Clinical malaria cases identified by passive case detection were responsible for only 1% of mosquito infections; if the definition of clinical malaria included infected individuals identified by community surveys with a history of fever in the preceding week, the contribution of clinical cases to mosquito infections increased to 16%.

**Conclusions:**

In eastern Gambia, malaria transmission is maintained by asymptomatic malaria-infected individuals, mostly adults, adolescents and school-age children, while clinical cases are comparatively less important for transmission.

## Highlights

• In the examined low transmission area, seasonal malaria chemoprevention protected young children from clinical malaria.
• Asymptomatic infections, often with low parasite densities, are more frequent than clinical malaria episodes.
• Most onward transmission to mosquitoes (84-99%)is due to asymptomatic malaria infections.

## Background

The significant decline of the global malaria burden observed since 2000 raised the hope for a malaria-free world.^1^ However, progress has stalled since 2015,^2^ calling for new tools and strategies to further reduce transmission.

In The Gambia, following the scale-up of preventive interventions such as long-lasting insecticide-treated nets (LLINs) and indoor residual spraying (IRS), and of prompt diagnosis and treatment with artemisinin-based combinations, the malaria burden in the last decades has significantly declined.^3^, ^4^ Nevertheless, despite a high coverage of control interventions, malaria transmission has not been interrupted,^3^ jeopardising the national goal of achieving malaria elimination by 2030.^5^ Therefore, to further improve control efforts and identify new control interventions, understanding residual transmission where coverage of control interventions is high becomes extremely important.^3^, ^6^, ^7^

Malaria vectors become infected when ingesting mature *Plasmodium* gametocytes (sexual forms of the malaria parasite circulating in human blood) that, after a complex cycle, will make the mosquito infectious to humans upon a subsequent bite. Gametocytes are found in both symptomatic and asymptomatic individuals. Asexual parasite densities are usually high in malaria patients (symptomatic malaria) and can be diagnosed by microscopy or Rapid Diagnostic Tests. Asymptomatic infected individuals (asymptomatic carriers) instead have low parasite densities, some of them detectable by microscopy but others only by more sensitive molecular methods (sub-microscopic infections).^8^ Gametocyte densities are generally higher in asymptomatic individuals^9^ and can persist for weeks or even months.^10^ Where transmission is high or moderate, microscopy-positive, asymptomatic carriers represent an important proportion of the human infectious reservoir.^11^, ^12^ However, as transmission declines, the proportion of asymptomatic carriers with parasite densities below the microscopy detection limit increases.^13^, ^14^ Moreover, the contribution of these sub-microscopic carriers to residual transmission is unclear. Indeed, this is important to establish as it may inform the control strategies to be implemented. If asymptomatic carriers with parasite densities below the microscopy detection threshold contribute significantly to residual transmission, mass screening and treatment with highly sensitive diagnostic tests may further reduce malaria transmission. Here, we investigated the relative contribution of symptomatic and asymptomatic infected individuals to the infectious reservoir in an area of low malaria transmission in The Gambia.

## Methods

### Study site and participants

This is an observational cohort study carried out during the 2019 malaria transmission season (June 2019 to March 2020). Eight villages with a population of 200–600 inhabitants were selected in Upper River Region (URR), The Gambia (Table 1). In this area, coverage of LLIN and IRS was high, 96.8% and 76.9%, respectively.^3^, ^4^ Seasonal malaria chemoprevention (SMC) in children below 5 years of age started in 2014, with a coverage of 86% in 2015 and 81% in 2016.^4^ In 2017, PCR-determined *P. falciparum* prevalence varied between 1.2% to 40.2%.^5^, ^15^

**Table 1:** Characteristics of malaria transmission in the study villages.

Parameters	Njayel	Banni Kunda/Temanto	Sare Wasa/Talito Luntang	Sare Samba Tacko	Madina Samba Sowe	Sotuma Sainey Kandeh	Sare Demba Dardo/Sare Biram	Musanding Kunda	Overall
Baseline									
Population	380	154	301	211	196	235	261	237	1975
Sex									
Female	206/380 (54.2)	91/154 (59.1)	170/299 (56.9)	119/211 (56.4)	120/196 (61.2)	138/235 (58.7)	139/261 (53.3)	132/237 (55.7)	1115/1973 (56.5)
Age groups									
<5 years	67/379 (17.7)	27/154 (17.5)	50/297 (16.8)	32/205 (15.6)	43/194 (22.2)	36/235 (15.3)	49/256 (19.1)	51/231 (22.1)	355/1951 (18.2)
5–15 years	147/379 (38.8)	69/154 (44.8)	116/297 (39.1)	88/205 (42.9)	73/194 (37.6)	79/235 (33.6)	89/256 (34.8)	85/231 (36.8)	746/1951 (38.2)
≥16 years	165/379 (43.5)	58/154 (37.7)	131/297 (44.1)	85/205 (41.5)	78/194 (40.2)	120/235 (51.1)	118/256 (46.1)	95/231 (41.1)	850/1951 (43.6)
Parasite prevalence at baseline (start of season = July 2019)									
Overall age group	9/378 (2.4)	14/153 (9.2)	3/291 (1.0)	8/200 (4.0)	5/181 (2.8)	10/233 (4.3)	41/252 (16.3)	29/227 (12.8)	119/1915 (6.2)
<5 years	1/67 (1.5)	0/27 (0.0)	1/48 (2.1)	0/31 (0.0)	2/40 (5.0)	1/36 (2.8)	5/48 (10.4)	5/49 (10.2)	15/346 (4.3)
5–15 years	3/147 (2.0)	6/69 (8.7)	0/115 (0.0)	6/87 (6.9)	0/68 (0.0)	2/78 (2.6)	15/88 (17.0)	14/84 (16.7)	46/736 (6.2)
≥16 years	5/164 (3.0)	8/57 (14.0)	2/128 (1.6)	2/82 (2.4)	3/73 (4.1)	7/119 (5.9)	21/116 (18.1)	10/94 (10.6)	58/833 (7.0)
Parasite prevalence at the end of transmission season (January–February 2020)									
Overall age group	13/347 (3.7)	22/149 (14.8)	8/210 (3.8)	9/219 (4.1)	6/186 (3.2)	5/150 (3.3)	17/233 (7.3)	13/162 (8.0)	93/1656 (5.6)
<5 years	1/57 (1.8)	0/24 (0.0)	1/23 (4.3)	0/31 (0.0)	0/36 (0.0)	0/23 (0.0)	2/40 (5.0)	4/31 (12.9)	8/265 (3.0)
5–15 years	6/144 (4.2)	9/68 (13.2)	5/111 (4.5)	3/98 (3.1)	0/70 (0.0)	1/64 (1.6)	7/91 (7.7)	4/63 (6.3)	35/709 (4.9)
≥16 years	6/146 (4.1)	13/57 (22.8)	2/76 (2.6)	6/90 (6.7)	6/80 (7.5)	4/63 (6.3)	8/102 (7.8)	5/68 (7.4)	50/682 (7.3)
Clinical incidence of malaria by PCD* (incidence rate [per 100 person-months of follow-up]; total clinical cases/total person-months of follow-up)									
Overall age group	1.67 (38/2274)	1.30 (12/924)	0.51 (9/1782)	0.41 (5/1230)	3.87 (45/1164)	0.28 (4/1410)	3.12 (48/1536)	0.72 (10/1386)	1.46 (171/ 11,706)
<5 years	1.49 (6/402)	0.62 (1/162)	0.00 (0/300)	0.00 (0/192)	1.16 (3/258)	0.00 (0/216)	1.70 (5/294)	0.98 (3/306)	0.85 (18/2130)
5–15 years	2.72 (24/882)	1.93 (8/414)	0.86 (6/696)	0.38 (2/528)	4.34 (19/438)	0.63 (3/474)	5.43 (29/534)	0.98 (5/510)	2.14 (96/4476)
≥16 years	0.81 (8/990)	0.86 (3/348)	0.38 (3/786)	0.59 (3/510)	4.91 (23/468)	0.14 (1/720)	1.98 (14/708)	0.35 (2/570)	1.12 (57/5100)

Note: Few individual records were not complete, and sex or age was not recorded, and adjusted for further analysis.

* PCD = passive case detection was done from August 2019 to January 2020.

In the study villages, malaria prevalence by qPCR in 2017 ranged between 1.2% and 15.4%.^16^ Prior to recruitment, the study team met with the village leaders to explain the study and obtain permission to implement it. Residents willing to participate were asked to provide a written informed consent. Parents/guardians provided a written informed consent for their children, who were also asked to provide their assent if aged 12 years or older. The study protocol received ethical approval from The Gambia Government/MRC Joint Ethics Committee and the Ethics Committee of the London School of Hygiene and Tropical Medicine (ref. 16642).

#### Data collection

Two cross-sectional surveys were implemented in July 2019 and January 2020, at the beginning and the end of the malaria transmission season, respectively. During these surveys, all participants in the study villages had their forehead temperature measured, and a blood sample collected by finger prick for molecular analysis. In case of fever and a measured forehead temperature of ≥37.5 °C with thermometer (Thermofocus model 01500A3), a Rapid Diagnostic Test (RDT) (SD BIOLINE Malaria Ag Pf, Standard Diagnostics Inc.) was done. If positive, treatment with artemether lumefantrine (AL), the first-line treatment in The Gambia,^17^ was administered.

Between August 28th, 2019, and January 28th, 2020, a passive malaria case detection (PCD) system was set up in each study village. Patients who presented with fever and/or history of fever within the last 24 h and without any other obvious illness had an RDT done by either a study nurse or a village health worker (VHW). Clinical malaria was defined as fever at visit or history of fever in the last 24 h and a positive RDT. In these patients, before administration of AL treatment, a blood sample was collected by finger prick in EDTA microtainer and RNA protect reagent in Eppendorf tubes (250–400 µL). Moreover, a few drops of blood were collected on filter paper for dried blood spots (DBS).

Besides PCD and the two cross-sectional surveys at the beginning and the end of the study, two community surveys were implemented during the malaria transmission season, at 8–10-week intervals (30th August to 11th November and 12th November to 16th January). These surveys consisted of collecting blood samples from all residents that were analysed within the same day by qPCR varATS to detect *P. falciparum* infections. If the qPCR result was positive, an additional venous blood sample (5–6 mL) was collected within 48 h for mosquito feeding assays and parasite and gametocyte quantification.

#### Laboratory analysis

To determine malaria prevalence at the beginning and at the end of the malaria transmission season, DBS from the two cross-sectional surveys were analysed by *var* gene acidic terminal sequence (*varATS*) PCR.^18^ For *varATS* PCR-positive samples, 100 µL of blood in RNA preservative (RNAprotect Cell Reagent; Qiagen, Hilden, Germany) were used for automatic extraction of parasite nucleic acids and analysis by 18S quantitative PCR (qPCR) (detection limit of 22 parasites/mL^19^) and by reverse-transcriptase qPCR (targeting male PfMGET and female CCp4 mRNA) for gametocytes; to estimate gametocyte prevalence, a limit of detection of 0.1 gametocytes/µL was used.^20^ The primer and probe sequences are described in Supplementary Table 1.

#### Direct membrane feeding assays

Direct membrane feeding assays (DMFA) were performed on a subset of patients with clinical malaria identified by PCD, and on asymptomatic *Plasmodium* infected carriers identified during the two community surveys. The number of individuals undergoing DMFA depended on the insectary capacity and no formal participants selection was done.

For mosquito feeding experiments, venous blood samples drawn in lithium heparin vacutainers (BD, Franklin Lakes, NJ, USA) were stored in thermos flasks and transported to the insectary within 4 h, as previously validated.^21^ Blood was offered to three cups of 40 lab-reared *Anopheles coluzzii* mosquitoes via three glass feeders filled with 0.5 mL of blood each.^22^ On day 7–8 post feeding, mosquitoes were dissected in 0.5% mercurochrome and examined by two independent microscopists for the presence of oocysts in the mosquito midguts.

#### Entomological surveys

To estimate natural exposure to malaria vectors, monthly entomological surveys were carried out for 4 nights per village (7 PM-7 AM) using indoor Centers for Disease Control and Prevention light-traps (CDC-LT) in six randomly selected houses. In each village, additional indoor-outdoor human landing catches (HLC) for three consecutive nights were performed monthly in four randomly selected houses (7 PM to 7 AM). Mosquito specimens were morphologically identified after each collection night using a pictorial key^23^ and a stereo microscope, and individually stored in tubes with silica gel for molecular analysis. *Anopheles gambiae* (*s l*) head and thorax samples were used for the detection of *P. falciparum* circumsporozoite protein (CSP) by ELISA.^24^

#### Statistical analysis

Incidence of clinical malaria was estimated by dividing the number of confirmed cases by the person-months of follow-up. Malaria prevalence was calculated by dividing the number of *varATS* PCR-positive samples by the number of samples analysed. Parasite density estimates were based on 18S qPCR. A Mann-Whitney U test was used to compare parasite or gametocyte densities between clinical malaria patients and asymptomatic carriers. Parasite and gametocyte densities were log-transformed and modelled using a Gaussian distribution. The association between parasite density (18S qPCR) and gametocyte density (PfMGET + CCp4) was determined separately for malaria patients and asymptomatic carriers using mixed effects linear regression models with log-transformed parasite and gametocyte densities and a random person-effect.

The association between the proportion of infected mosquitoes after DMFA and gametocyte density was determined using a generalised linear model assuming a binomial distribution with a log-link for symptomatic and asymptomatic infections together. The contribution of symptomatic and asymptomatic microscopic infections (assigned using a qPCR threshold of ≥20 or ≥100 parasite/µL) and sub-microscopic (assigned as qPCR threshold of <20 or <100 parasites/µL) to the infectious reservoir was calculated by incorporating the proportion of these infections in the infected population and their average infectivity; these analyses including all detected infections have been previously described in detail and are included in the Appendix methods.^14^ The estimation of contributions of different age groups (younger than 5 years, 5–15 years, 16 years and older) to the infectious reservoir included all visits, including parasite-negative observations, following a similar approach (Appendix methods).

Entomological outcomes were summarised overall and by village. Mosquito density was computed using negative binomial regression with the number of mosquitoes as the outcome and trapping nights as the offset. The biting rate was estimated by dividing the number of mosquitoes captured by the number of capturers and days. The sporozoite rate was calculated as the number of CSP-positive mosquitoes divided by the total number analysed for CDC-light traps and human landing catches (HLC). The monthly entomological inoculation rate (EIR) was estimated as the sporozoite rate multiplied by the average number of mosquitoes captured by CDC-LT multiplied by 30, while for HLC this was the product of sporozoite rate and biting rate multiplied by 30. The 95% confidence interval for the EIR was computed using bootstrapping.

All statistical analyses were performed in R (version 3.1.12) and Stata Version 17.0. Full details of the statistical methods are in the Supplementary Material.

## Results

The total population in the eight study villages was 2452. The first survey, in July 2019, included 1975 individuals (80.5% of the eligible population); the second survey, in February 2020, included 1726 individuals (70.4%).

Malaria prevalence in July was 6.2% (119/1915) and was slightly lower in children <5 than in older children and adults (Table 1). There was substantial heterogeneity between villages as prevalence varied between 1.0% (3/291) and 16.3% (41/252). Prevalence of infection in January to February was 5.6%, with similar distribution between age groups and heterogeneity between villages as in the earlier survey (Table 1). Overall, during community surveys, 35.3% (36/102) of survey participants reported fever in the last 24 h and 52.9% (54/102) in the last 7 days (Table 2).

**Table 2:** Mosquito feeding assay outcomes by the number of feeds done, mosquitoes dissected, and infectious feeds stratified by symptomatic and asymptomatic infections.

	Number of feeds	Number of mosquitoes dissected	Proportion of infectious individuals % (n/N)	Proportion of infected mosquitoes % (n/N)
Infection types				
Overall	142	14,176	4.92 (7/142)	1.60 (227/ 14,176)
Symptomatic (PCD)	40	3961	0 (0/40)	0 (0/3943)
Asymptomatic (CS)	102	10,215	6.86 (7/102)	2.22 (227/ 10,215)
No fever reported	46	4726	4.35 (2/46)	0.87 (42/4816)
Fever during the last 24 h	36	3464	8.33 (3/36)	3.98 (138/3464)
Fever during the last 7 days	54	5289	9.26 (5/54)	3.49 (185/5289)
Age group				
<5 years	2	195	100 (2/2)	37.17 (71/195)
5–15 years	70	6910	1.42 (1/70)	0.67 (46/6892)
≥16 years	69	6963	5.79 (4/69)	1.58 (110/6963)

Asymptomatic infections were further subdivided as follows: Individuals with no reported fever, fever last 24 h and fever last 7 days (inclusive of fever last 24 h).

PCD, passive case detection; CS, community surveys; n, number of individuals and number of mosquitoes infected; N, total number of individuals included, and total number of mosquitoes dissected; %, percentage.

Between August 2019 and January 2020, the overall incidence of clinical malaria by PCD was 1.46/100 person-months (171/11,706 person-years) (range 0.85–2.14/100 person-months). Incidence was the highest among the 5–15 years age group (2.14/100 person-months, 96/4476 person-years), followed by adolescents and adults (1.12/100 person-months, 57/5100 person-years) (Table 1). Incidence of clinical malaria varied between study villages, between 0.28/100 person-months and 3.87/100 person-months (Table 1).

**Fig. 1:**
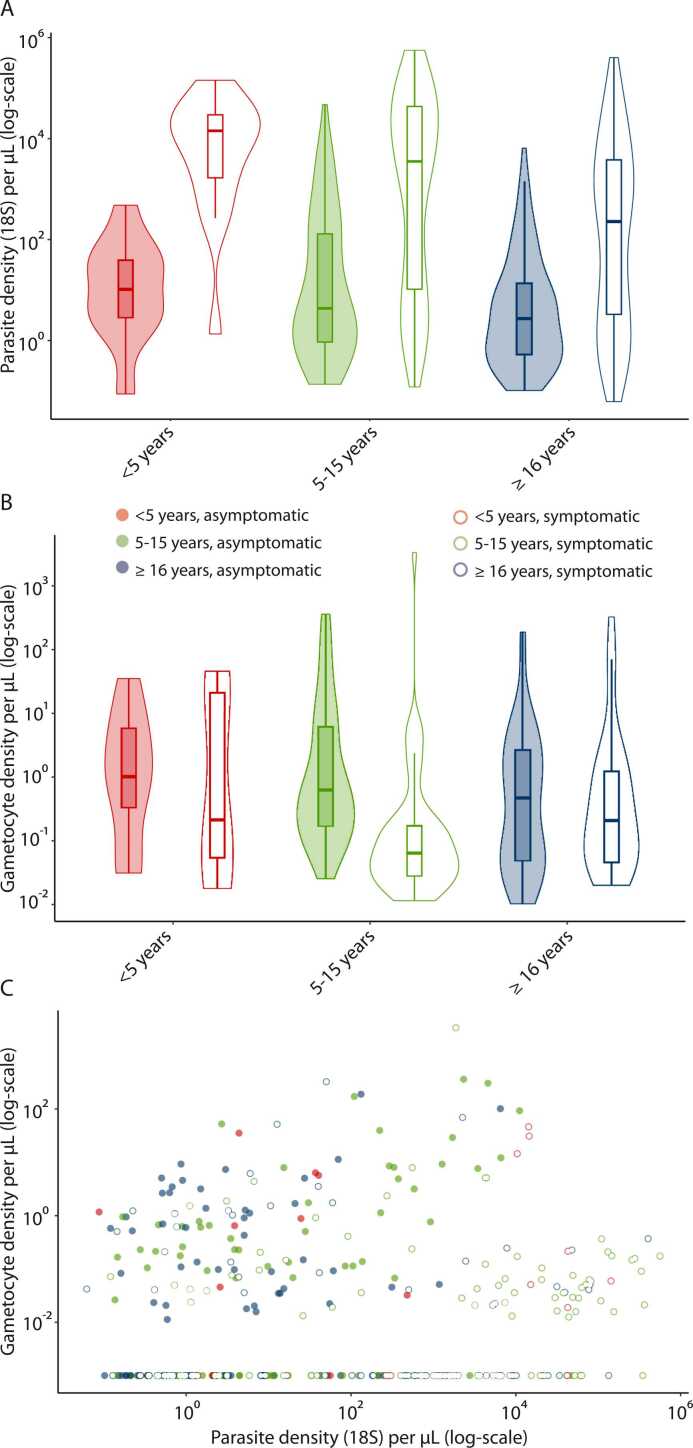
Parasite and gametocyte densities. (A) Parasite density among qPCR-positive asymptomatic (n = 163) and symptomatic individuals (n = 190) by age groups. (B) Gametocyte density among qRT-PCR-positive asymptomatic and symptomatic individuals across all surveys by age groups. (C) Parasite density by gametocyte density in asymptomatic and symptomatic individuals across all surveys by age groups. Abbreviations: qPCR, quantitative Polymerase chain reaction; qRT-PCR, quantitative reverse transcriptase polymerase chain reaction.

Parasite density was estimated by 18S qPCR in 134 samples from Plasmodium-infected individuals (positive by *varATS* PCR) identified during the community surveys. The median parasite density was significantly lower in asymptomatic (3.67 parasites/µL, interquartile range [IQR], 0.77–39.52) than symptomatic individuals (1459.0 parasites/µL, IQR 7.86–21,172.9, P < 0.0001) and this trend was seen in all age groups (Fig. 1A). Gametocytes were detected in 37.2% (80/215) of asymptomatic infections and 24.7% (50/202) of symptomatic infections (Supplementary Table 2). Their median density was significantly higher in asymptomatic (0.63 gametocytes/µL, IQR 0.10–3.39) than in symptomatic infections (0.08 gametocytes/µL, IQR 0.04–0.53, P < 0.001) (Fig. 1B, Supplementary Table 2). Gametocyte density was significantly associated with parasite density in asymptomatic (β = 0.40; P < .0001) but not in symptomatic infections (β = 0.03; P = 0.61) (Fig. 1C).

One hundred forty-two membrane feeding experiments were performed, 40 on malaria patients identified by PCD and 102 on asymptomatic malaria-infected individuals identified during the community surveys (Table 2). These experiments involved 17,040 mosquitoes, of which 94.0% (16,018/17,040) fed successfully and 88.5% (14,176/16,018) survived until dissection. Only seven individuals, all of them from community surveys, were able to successfully infect at least one mosquito. The mean number of oocysts per infected mosquito was 5.70 [range 1–22.8]. Among these individuals detected during community surveys, those with a history of fever in the past 24 h or past 7 days tended to be more infectious to mosquitoes than those without any symptoms reported in this period (Table 2).

**Fig. 2:**
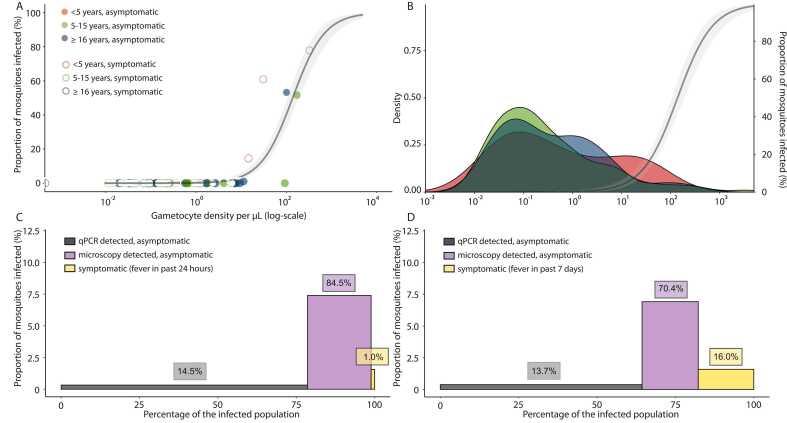
Relationship between gametocyte density determined by qRT-PCR and percentage of mosquitoes infected and subgroups contribution to the infectious reservoir. (A) The relationship between gametocyte density and the proportion of mosquitoes infected. The line represents the best-fitted association; the shaded area represents the 95% CI. (B) Gametocyte density among qRT-PCR positive individuals by age groups. The y-axis represents the density of a gametocyte concentration in the infected population, separated by age group. The line represents the best-fitted association between gametocyte density and the proportion of infected mosquitoes, and the shaded area is the 95% CI. (C) Contribution of different infection types (symptomatic vs. asymptomatic sub-microscopic and microscopic) in the infected population stratified by cut-off value of microscopy (<20 parasites/µL by 18S qPCR) in relation to fever last 24 h and (D) last 7 days. The contribution of asymptomatic submicroscopic and microscopic and symptomatic infections to the infectious reservoir was determined based on measured mosquito infection rates, if available, and, by imputing mosquito infection rates for samples with known gametocyte densities. Bars heights represent the proportion of infected mosquitoes, bar widths the proportion of each infection type in the infected population. Sample size corresponding to bar widths is described in the Appendix methods. The percentage indicated above each bar is the contribution of each infection type to the infectious reservoir. Abbreviations: qRT-PCR, reverse transcriptase quantitative polymerase chain reaction.

Gametocyte densities were strongly associated with the proportion of mosquitoes infected (β = 2.81; P < 0.0001) (Fig. 2A and B); one individual who infected a single mosquito but was gametocyte negative by RT-qPCR was excluded from this analysis but included in the infectious reservoir plot (Appendix methods). The contribution of asymptomatic malaria-infected individuals and symptomatic infections to the infectious reservoir was estimated using the measured mosquito infection rates if available and, if only gametocyte density data were available, by imputing mosquito infection rates from gametocyte density using the association mentioned above (Fig. 2A).

Most of the human infectious reservoir (84.5%) was represented by asymptomatic individuals (not reporting fever in the previous 24 h) with a parasite density of at least 20/µL while asymptomatic individuals with lower parasite densities (<20/µL) represented 78.6% of the infected human population and constituted 14.5% of the human infectious reservoir (Fig. 2C). Symptomatic infections defined as malaria patients with fever reported in the past 24 h identified during the community surveys constituted only 1% of the human infectious reservoir (Fig. 2C, Supplementary Table 2). Similar results were obtained when increasing the threshold for sub-microscopic infection from 20/µL to 100/µL (Supplementary Figure 1). However, when including among symptomatic individuals all those with a history of fever in the past 7 days, the contribution of symptomatic malaria to the infectious reservoir increased to 16.0% (Fig. 2D).

**Fig. 3:**
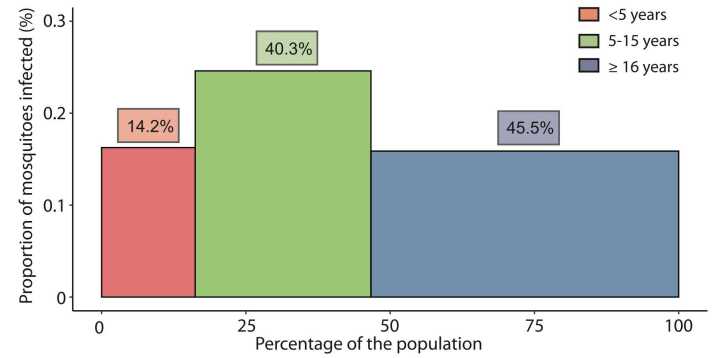
Contribution of different age categories to the human infectious reservoir at the population level. Contribution to the infectious reservoir of asymptomatic infections was estimated based on imputing gametocyte densities among asymptomatic samples with known parasite densities and imputing the proportion of infected mosquitoes among asymptomatic samples with mosquito feeding results and known gametocyte densities. Bar heights represent the proportion of infected mosquitoes; the bar widths come from Gambia (United Nations) population statistics of 2019 and represent the proportion of each age category among the total population, as described in the Appendix methods. The percentage given above represents their proportion contributing to the infectious reservoir in the entire population.

We finally investigated the infectious reservoir in the total population, including parasite-negative visits, for different age categories (Appendix methods). Individuals aged 16 years or older were responsible for an estimated 45.5% of the infectious reservoir, followed by children 5–15 years old (40.3%) and children <5 years (14.2%) (Fig. 3).

During the study period, 6970 mosquito specimens were collected, 82.4% (5746/6970) with HLC and 17.6% (1224/6970) with CDC-LT. The mean vector density per trapping night was 1.70 (range 0.69–4.22) by CDC-LT and 11.97 (4.16–34.42) for HLC indoor and outdoor combined (Supplementary Table 3). The mean human biting rate was 8.98 (3.12, 25.81) per person night (Supplementary Table 3). The sporozoite rate was 0.79% (9/1143) for CDC-LT and 0.39% (11/2819) for HLC. Therefore, the monthly EIR was 0.40 (0.00, 0.83) for CDC-LT and 0.35 (0.00, 0.47) infected bites per person per month for HLC.

## Discussion

This study assessed the human infectious reservoir in an area of low transmission intensity in The Gambia and observed that most mosquito infections resulted from asymptomatic parasite carriers. None of the clinical malaria cases identified by PCD were able to infect mosquitoes. Conversely, all successful infections occurred with blood from study participants from community surveys, especially if they reported fever in the last 24 h or even last week.

The contribution of asymptomatic infections to residual transmission and the necessity of interventions targeting these infections remains unclear. Such evidence is available in areas of high transmission where asymptomatic infections are important contributors to transmission.^14^, ^25^, ^26^, ^27^ However, there are few studies of this kind in areas of low transmission,^12^, ^14^, ^25^ possibly because of the logistical complexities to assess transmission potential from the sparse infections that are often of very low parasite density.^8^, ^12^ The current study was implemented in eastern Gambia, and area of low seasonal transmission, although transmission intensity is even lower in the rest of the country. In this area, most onward transmission is caused by asymptomatic individuals with parasite densities that are deemed detectable by microscopy.^3^, ^28^ In this study, we arbitrarily defined such detection limit at 20 parasites per microlitre, an extremely low threshold for routine microscopy that usually examines blood slides by counting the number of parasites per 200 white blood cells; detecting 1 parasite would correspond to a density of about 40 parasites per microlitre, a higher threshold than the one we arbitrarily defined. However, even if the detection threshold is increased to 100 parasites per microlitre, i.e., 2–3 parasites per 200 white blood cells on the slide, asymptomatic individuals are still the most important contributors to residual transmission. Among asymptomatic parasite carriers, those with an infection detectable by microscopy are most infectious, whereas those detectable only by molecular methods represent 13.7%–15.5% of the infectious reservoir, as reported by other studies.^29^, ^30^

Quantifying the relative importance of symptomatic infections for malaria transmission was challenged by the observation that symptoms were regularly reported in cross-sectional surveys by individuals who did not seek treatment. During the community surveys, about half of the infected individuals reported having had fever in the previous few days. The cause of this reported fever is unknown and may, at least partially, be attributable to infections other than malaria. Therefore, besides the fact that malaria patients are probably not adequately managed, clearly defining clinical malaria versus asymptomatic infection may be problematic. Acknowledging this complexity of defining clinical malaria,^31^ we examined the transmissibility of infections using different definitions of clinical/symptomatic malaria based on history of fever within the last 24 h or within the last week. The relative contribution of clinical malaria to the infectious reservoir was greatly influenced by broadening its definition to include individuals with a history of fever within the previous 7 days who did not actively seek treatment. With this inclusive definition, up to 16% of the infectious reservoir was attributable to (mild) symptomatic infections. Nevertheless, the vast majority of mosquito infection events occurred from asymptomatic infections, as also observed in other areas of low transmission, such as Uganda^14^ and Ethiopia.^12^

These observations should be considered when choosing the control interventions most appropriate for this context. Preventing onward transmission from clinical malaria cases, which may be optimised by combining single low-dose primaquine with an ACT as first-line treatment, is likely to have a very limited impact given their small contribution to the infectious reservoir. This seems supported by a recent study from high endemic Burkina Faso where repeated fever screening and treatment of clinical cases had a marginal effect on the human infectious reservoir.^32^ Mass screening with RDT, whose detection limit threshold is around 200 parasites per microlitre^33^, ^34^ and will miss many gametocyte-positive infections, may only have a large impact on the infectious reservoir when sustained through peak and low malaria seasons and will still not completely empty the reservoir. More sensitive field-deployable assays, potentially combined with efficacious treatment with single low-dose primaquine may be the most promising approach to target the infectious reservoir with (repeated) rounds of screening and treatment.

Our findings are, as all assessments of the human infectious reservoir, context-specific. Incidence of clinical malaria was low in our study setting, i.e., 1.46 episodes per 100-person months, due to the low exposure to infectious bites as shown by the low EIR. As expected, both incidence and prevalence of infection tended to be lower in children aged <5 years, probably reflecting the efficacy of SMC.^35^, ^36^ However, the prevalence of infection in July, before the implementation of SMC, was similar to the prevalence in January-February. This last survey was implemented after the end of the transmission season and a few months after the last SMC round was implemented, when the prophylactic effect of SMC would have waned. The difference in malaria prevalence between the first and the second survey, and between the <5 years age group and the other age groups, may have been more pronounced if the survey had been done at peak transmission, in November. SMC is also likely to have impacted the relative contribution of different age groups to the infectious reservoir by reducing infection rates and the duration of infections in the youngest age group.^4^, ^15^ Our results are consistent with observations from other countries reporting the significant contribution of school-age children (5–15 years) to the infectious reservoir.^14^, ^37^ Nevertheless, contrary to previous reports,^12^, ^14^, ^25^, ^27^ in our setting, adolescents and adults represent an important proportion of the human infectious reservoir and thus should be considered when deciding which groups to target for control interventions. In The Gambia, any intervention aiming at reducing the human infectious reservoir should target the whole population to have maximum impact.

This study has a few shortcomings. The first shortcoming is that our conclusions are based on a limited number of infectious individuals. While conducting 142 experiments, only 7 resulted in mosquito infections. Infectivity and mosquito infection rates were strongly associated with gametocyte densities and this association was similar to that recently reported in an area of low malaria transmission intensity in Uganda^14^ (Supplementary Figure 2). We thus feel confident that our imputation of mosquito infection rates for 335 observations where gametocyte density data were available but no DMFA was performed is informative. A second limitation is the low number of children <5 years recruited for the DMFA experiments. This was due to the small number of infections observed in this age group and may have resulted in lower precision, but is unlikely to affect overall conclusions.^27^ Thirdly, our repeated cross-sectional surveys still leave questions unanswered regarding the natural history of infections, fluctuations in parasite and gametocyte densities and the (transient) nature of malaria symptoms.^14^

In conclusion, malaria transmission in The Gambia is mainly maintained by asymptomatic malaria-infected individuals, mostly older children (5–15 years old) and individuals aged 16 years and above. Targeting only clinical malaria cases is likely to have a limited impact on transmission since their contribution to transmission is relatively small, even if the definition of clinical malaria is broadened to include a history of fever in the previous week. This calls for interventions able to target efficiently asymptomatic carriers. There are currently no field-deployable diagnostic tests able to detect infections of low to very low parasite density. RDT will probably miss an important proportion of infected individuals, while the capacity of microscopy to screen a large number of samples is limited. Until such a diagnostic test becomes available, mass screening and treatment cannot be considered as an operationally attractive, effective intervention for further reducing transmission.

## Sources of funding

This work was supported by the Bill & Melinda Gates Foundation [grant INDIE OPP1173572] to TB, CD and UA. SLB and TB are further supported by a fellowship from the European Research Council (ERC-CoG 864180 QUANTUM). T.B. is further supported by a fellowship from the Netherlands Organization for Scientific Research (Vici fellowship NWO 09150182210039).

## Author contribution

HMS, SLB: Investigation, methodology, writing of original draft, formal analysis, reviewing and editing. SLB, SS, NIM and MO: Data analysis. AA, PMG, LJ, MMC, EAJ, KL, BE, LC, MON, BC, AKN, AN and AE: Investigation, methodology, reviewing and editing. UDA, CD, MM, TB: Conceptualisation, funding acquisition, investigation, methodology, project administration, supervision, validation, visualisation, reviewing and editing. All authors read and approved the final manuscript.

## Declaration of Competing Interest

The authors declare that they have no known competing financial interests or personal relationships that could have appeared to influence the work reported in this paper.

## Footnotes

Appendix A Supplementary data associated with this article can be found in the online version at doi:10.1016/j.jinf.2025.106507.
