# Supplementary material for: Asymptomatic school children and adults are important for the human infectious reservoir for Plasmodium falciparum malaria in an area of low endemicity in The Gambia
Source: J Infect. 2025 Jul;91(1):106507. doi: 10.1016/j.jinf.2025.106507 (PMC12170349; doi:10.1016/j.jinf.2025.106507)
Supplement: Supplementary file 1 — Supplementary material [file mmc1.docx]

Supplemental material for Soumare *et al.,*

**Title:** Asymptomatic school children and adults are important for the human infectious reservoir for Plasmodium falciparum malaria in an area of low endemicity in the Gambia

**Supplementary figure 1**. **Fitted lines for the association of gametocyte density with percentage of mosquitoes infected in The Gambia and Uganda.** The relationship between gametocyte density and the proportion of mosquitoes infected, dots represent the data points from the current study conducted in The Gambia. Fitted lines represent the best fitted association for current data from The Gambia (grey line) and previously published data from Uganda (green line), shaded areas represent the 95% CIs. Data from Uganda is from a cohort study in a low endemic setting in Eastern Uganda [14].

**Supplementary figure 2**. Contribution of different infection types (symptomatic vs. asymptomatic sub-microscopic and asymptomatic microscopic) in the infected population stratified by cut-off value of microscopy (<100 parasites/µL by 18S qPCR) in relation to fever in the last 24 hours (A) and last 7 days (B). The contribution of asymptomatic submicroscopic, asymptomatic microscopic and symptomatic infections to the infectious reservoir was determined based on measured mosquito infection rates if available and, if unavailable, by imputing mosquito infection rates for samples with known gametocyte densities. Bar heights represent the proportion of infected mosquitoes, bar widths the proportion of each infection types in the infected population. Sample size corresponding to bar widths is described in the appendix methods. The percentage indicated above each bar is the contribution of each infection type to the infectious reservoir.

Supplementary Table 1: Primer and probe sequences. Primers used in varATS qPCR and female (CCp4) and male (PfMGET) gametocyte RT-qPCR

**
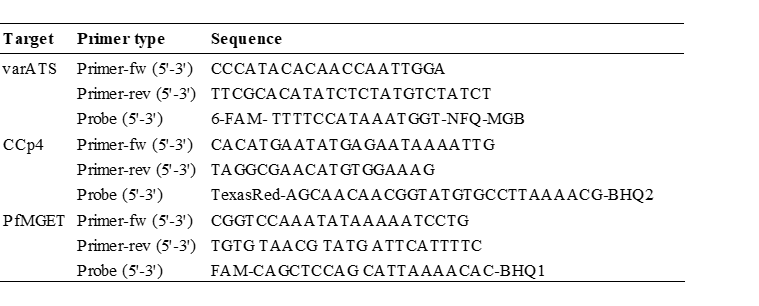
**

**Supplementary Table 2**: Prevalence of infected populations and infection characteristics.

Prevalence of symptomatic versus asymptomatic infections was determined based on community surveys visits with varATS qPCR data. A symptomatic infection was defined as a positive diagnostic test (varATS or RDT) with measured or reported fever in past 24 hours. Asymptomatic infection was defined as a positive varATS or positive 18S qPCR visit without fever in the past 24 hours. 20.5% (31/151) of asymptomatic infections with parasite densities quantified by 18S qPCR was defined as microscopy detectable when using a threshold of 20 parasites/µL by 18S qPCR; 79.5% (120/151) was detectable by qPCR only (i.e sub microscopic). Considering all asymptomatic visits in routine surveys (n = 247), the estimated proportion of microscopy versus qPCR detectable infections would be 1.59% and 6.11%, respectively. Median parasite density was calculated amongst 18S qPCR positive observations across all surveys. Gametocyte prevalence (>0.10 gametocytes/µL) was calculated amongst infections with parasite densities quantified by 18S across all surveys and median gametocyte density (in gametocytes/µL) was calculated amongst gametocyte positive observations.

| Infection type | Prevalence | Total parasite density  (IQR) | Gametocyte prevalence | Gametocyte density  (IQR) | Proportion infected mosquitoes  observed | Proportion infected mosquitoes  average | Contribution to the infectious reservoir |
| --- | --- | --- | --- | --- | --- | --- | --- |
| Symptomatic | 3.49% (112/3207) | 1459.0  (7.86-21172.9) | 24.7% (50/202) | 0.08  (0.04-0.53) | 2.45%  (138/5627) | 1.56% | 1.0% |
| Asymptomatic | 7.70% (247/3207) | 3.67  (0.77-39.52) | 37.2% (80/215) | 0.63  (0.10-3.39) |  |  |  |
| Microscopic  (≥ 20 parasites/µL) | 1.59% (51/3207) | 212.5  (57.2-890.5) | 58.0%  (29/50) | 4.88  (0.14-11.7) | 5.23%  (88/1683) | 7.39% | 84.5% |
| Sub microscopic  (< 20 parasites/µL) | 6.11% (196/3207) | 1.36  (0.45-4.09) | 30.9% (51/165) | 0.43  (0.09-0.95) | 0.04%  (1/2832) | 0.33% | 14.5% |

IQR: Interquartile range, uL microliter.

**Supplementary Table 3:** Vectors mean density, biting rate, sporozoite rate and entomological inoculation rate (EIR) in relation to collection methods and site per village and overall

| Village | Mosquito density | | | | Biting rate | | Sporozoite rate | | EIR | EIR |
| --- | --- | --- | --- | --- | --- | --- | --- | --- | --- | --- |
|  | CDC-LT | | HLC | | HLC | | CDC-LT | HLC | HLC | CDC-LT |
|  | Number mosquitoes/ trapping nights | Mean density per night (95%CI) | Number mosquitoes/ trapping nights | Mean density per night (95%CI) | Number mosquitoes/ Capturers | Mean density per night (95%CI) | Elisa positive/ Number tested | Elisa positive/ Number tested | Rate (95% CI) | Rate (95% CI) |
| Banni kunda/Temanto | 482/90 | 5.36  (1.86, 15.44) | 957/60 | 15.95  (7.19, 35.36) | 957/240 | 11.96  (5.40, 26.52) | 3/402 (0.75) | 1/528 (0.19) | 0.23 (0.00, 0.63) | 1.20  (0.00, 2.79) |
| Madina Samba Sowe | 78/90 | 0.87  (0.42, 1.79) | 997/60 | 16.62  (4.92, 56.08) | 997/240 | 12.46  (3.69, 42.06) | 3/78 (3.85) | 4/502 (0.80) | 0.99 (0.00, 2.51) | 1.00  (0.00, 3.33) |
| Musanding Kunda | 92/90 | 1.02  (0.40, 2.64) | 489/60 | 8.15  (2.88, 23.07) | 489/240 | 6.11  (2.16, 17.30) | 0/92 (0.00) | 0/288 (0.00) | 0.00 (0.00, 0.00) | 0.00  (0.00, 0.00) |
| Njayel | 54/90 | 0.60  (0.21, 1.70) | 250/60 | 4.17  (0.24, 72.58) | 250/240 | 3.13  (0.18, 54.44) | 0/53 (0.00) | 0/86 (0.00) | 0.00 (0.00, 0.00) | 0.00  (0.00, 0.00) |
| Sare Samba Tacko | 90/90 | 1.00  (0.34, 2.98) | 1557/60 | 25.95  (6.88, 97.92) | 1557/240 | 19.46  (5.16, 73.44) | 1/90 (1.11) | 2/659 (0.30) | 0.59 (0.00, 1.04) | 0.33  (0.00, 1.67) |
| Sare demba dardo/Sare biram | 142/90 | 1.58  (0.48, 5.20) | 383/60 | 6.38  (1.43, 28.40) | 383/240 | 4.79  (1.08, 21.30) | 0/142 (0.00) | 2/192 (1.04) | 0.50 (0.00, 1.25) | 0.00  (0.00, 0.00) |
| Sare wasa/talito_  luntang | 30/90 | 0.33  (0.11, 1.04) | 309/60 | 5.15  (1.07, 24.84) | 309/240 | 3.86  (0.80, 18.63) | 0/30 (0.00) | 1/145 (0.69) | 0.27 (0.00, 0.85) | 0.00  (0.00, 0.00) |
| Sotuma Sainey Kandeh | 256/90 | 2.84  (0.85, 9.57) | 804/60 | 13.40  (4.72, 38.07) | 804/240 | 10.05  (3.54, 28.55) | 2/256 (0.78) | 1/419 (0.24) | 0.24 (0.00, 1.06) | 0.67  (0.00, 3.33) |
| Overall | 1224/720 | 1.70  (0.69, 4.22) | 5746/480 | 11.97  (4.16, 34.42) | 5746/1920 | 8.98  (3.12, 25.81) | 9/1143 (0.79) | 11/2819 (0.39) | 0.35 (0.00, 0.47) | 0.40  (0.00, 0.83) |

CDC-LT, center for disease control Light trap; HLC, human landing collection; EIR, Entomological inoculation rate. EIR (HLC) computed as (Elisa positive/Tested Elisa) * (Number of mosqutoes/Number of capturers/days)*30 ; EIR (CDC-LT) computed as (Elisa positive/Tested Elisa) * (Number mosquitoes/trapping nights)*30; The 95% CI of the EIR was estimated from percentile confidence intervals (CI) using the bootstrap

**Appendix methods:**

**Appendix methods: detailed statistical methods**

Statistical analyses were performed in Rstudio (version 4.1.3) and dplyr and ggplot2 packages were used for data manipulation and figure creation. For constructing the regression models, we used the lme4/lmerTest packages.

**Regression models for estimating associations**

To model the association between total parasite density (18S qPCR) and gametocyte density, generalized linear models were used for symptomatic and asymptomatic infections separately. Parasite density was considered a fixed effect, and individual specific random intercepts were incorporated. All continuous densities (*d*) were log transformed as log10(*d*+0.001), since densities could possibly be zero. If zero densities were not needed to be included in the model, the transformation was log10(*d*). For models with continuous responses, a gaussian distribution was assumed.

A generalized linear regression model was also used for modelling the effect of gametocyte density on the proportion of mosquitoes that became infected after a blood meal. The proportion of infected mosquitoes was set as the outcome variable, which was assumed to be derived from a binomial distribution with n = number of mosquitoes dissected, and a log-link function. The gametocyte density (Log10(*gam* + 0.001) was used as the explanatory variable and random intercepts were not included in this model. The model for estimating the proportion of infected mosquitoes was ran for symptomatic and asymptomatic infections together. The dataset consisted of 100 feeds with quantified gametocyte densities, one observation was excluded (IN_3_0900); this individual infected one mosquito but had no gametocytes detectable by qPCR. Exclusion of this individual influenced the P-value of the fitted line for gametocyte density versus proportion of infected mosquitoes (from P = 0.08 to P < 0.0001 upon exclusion) yet changed the estimate (β) only marginally (from β = 2.71 to β = 2.81). The observation was excluded for modeling the association between gametocyte density and the proportion of infected mosquitoes used for imputing the proportion of infected mosquitoes. The observation was included for estimating the contributions to the infectious reservoir.

**Contribution to the infectious reservoir by infection category**

The contribution of different infection categories (symptomatic, asymptomatic microscopy-detected, and asymptomatic PCR-detected) to the infectious reservoir was estimated as the proportion of the infected population per category relative to the transmissibility to mosquitoes in each category. First, the proportion of infections (prop) in each infection category was calculated amongst those that were parasite positive. For symptomatic *P. falciparum* infections, we used the clinical incidence from passive case detection (1.46 cases per 100 person-months) and assumed that an untreated symptomatic infection will last 14 days, giving 20.44 clinical days for 100 person-months and a likelihood of 20.44/3000*100 = 0.68% for someone to have clinical malaria at any day. The proportion of symptomatic infections was then calculated based on the number of observations in the routine survey with parasite data (18S qPCR). A total of 252 observations with 18S parasite quantification were included in the routine survey, giving a proportion of 252*0.0068 = 1.714 clinical cases. For sensitivity purposes, a less conservative definition of symptomatic cases was used for the construction of a second and separate infectious reservoir. In addition to the 1.714 estimated cases with fever in the past 24 hours, an additional 25 cases were added that were parasite-positive and had self-reported fever in the past 7 days during the routine visits (but not in the past 24 hours). For calculating the proportion of asymptomatic infections detected by microscopy versus by qPCR, we used a threshold of ≥20 parasites per µl by qPCR (18S) in routine survey visits. To assess the effect of this threshold for microscopy-detectable infections, we also constructed a separate infectious reservoir plot with a threshold of ≥100 parasites per µl instead.

Then, from symptomatic and asymptomatic samples with feeding data, we calculated the proportion of infected mosquitoes in that infection category (PI). For samples across all surveys without feeding data, the association between gametocyte density and the proportion of infected mosquitoes was used to calculate the proportion of infected mosquitoes. Then, the contributions to the infectious reservoir for each infection category was calculated by multiplying the mean proportion of infected mosquitoes (Prop_inf) of each infection category by the proportion of that infection category (prop) in the parasite positive population. Then, the relative contribution to the infectious reservoir of each infection category (PIprop) was given by dividing the proportion of infected mosquitoes (PI) by the sum of the proportion of infected mosquitoes in each category (sumPI).

**Table 1:** parameters for infectious reservoir plot with cut-off for microscopy as ≥ 20 parasites /µl.

| Category | N | n | Prop | Prop_inf | PI | sumPI | PIprop | fever |
| --- | --- | --- | --- | --- | --- | --- | --- | --- |
| Asymptomatic PCR-detected | 152.714 | 120 | 0.786 | 0.003 | 0.0026 | 0.0177 | 0.1452 | Fever 24h |
| Asymptomatic microscopy-detected | 152.714 | 31 | 0.203 | 0.074 | 0.0150 | 0.0177 | 0.8449 | Fever 24h |
| Symptomatic malaria | 152.714 | 1.714 | 0.011 | 0.016 | 0.0002 | 0.0177 | 0.0099 | Fever 24h |
| Asymptomatic PCR-detected | 150.714 | 97 | 0.644 | 0.004 | 0.0024 | 0.0176 | 0.1365 | Fever 7 days |
| Asymptomatic microscopy-detected | 150.714 | 27 | 0.179 | 0.069 | 0.0124 | 0.0176 | 0.7039 | Fever 7 days |
| Symptomatic malaria | 150.714 | 26.714 | 0.177 | 0.016 | 0.0028 | 0.0176 | 0.1595 | Fever 7 days |

**Table 2:** parameters for infectious reservoir plot with cut-off for microscopy as ≥ 100 parasites /µl.

| Category | N | n | Prop | Prop_inf | PI | sumPI | PIprop | fever |
| --- | --- | --- | --- | --- | --- | --- | --- | --- |
| Asymptomatic PCR-detected | 152.714 | 130 | 0.851 | 0.0034 | 0.003 | 0.0186 | 0.1551 | Fever 24h |
| Asymptomatic microscopy-detected | 152.714 | 21 | 0.138 | 0.1131 | 0.015 | 0.0186 | 0.8354 | Fever 24h |
| Symptomatic malaria | 152.714 | 1.714 | 0.011 | 0.016 | 0.0002 | 0.0186 | 0.0094 | Fever 24h |
| Asymptomatic PCR-detected | 150.714 | 107 | 0.710 | 0.0038 | 0.003 | 0.0180 | 0.1502 | Fever 7 days |
| Asymptomatic microscopy-detected | 150.714 | 17 | 0.113 | 0.1107 | 0.012 | 0.0180 | 0.6939 | Fever 7 days |
| Symptomatic malaria | 150.714 | 26.714 | 0.177 | 0.0158 | 0.003 | 0.0180 | 0.1559 | Fever 7 days |

**Contribution to the infectious reservoir by age category**

The proportion of infected mosquitoes in each age category weighted by the population proportion in that category was used to calculate the contribution to the infectious reservoir of each age category (<5 years, 5-15 years, >5 years). For symptomatic and asymptomatic samples with mosquito feeding data, the proportion of infected mosquitoes was calculated. For samples with gametocyte data but without a feed performed, the proportion of infected mosquitoes was imputed based on the relationship between gametocyte density and proportion of infected mosquitoes. For all *P. falciparum* negative visits (by varATS), the proportion of infected mosquitoes was specified as 0. We then estimated the average proportion of infected mosquitoes within each age category weighted by the proportion of the population in each age category (prop), which was based on UN population census data for the Gambia in 2019. The contribution to the infectious reservoir (PI) for each age category was calculated by multiplying the proportion in the population (prop) by the proportion of mosquitoes infected (Prop_inf) for each age category. Then, the relative contribution to the infectious reservoir of each age category (PIprop) was given by dividing the proportion of infected mosquitoes by the sum of all proportions of infected mosquitoes for each age category (sumPI).

**Table 3:** parameters for infectious reservoir plot of total human population including parasite negative observations, divided by age-category. N = total population size based on UN census data for the Gambia in 2019 (x 1000), n = population size for a given age group (x 1000), Prop = the proportion of a specific age group within the total population (= n/N).

| Category | N | n | Prop | Prop_inf | PI | sumPI | PIprop |
| --- | --- | --- | --- | --- | --- | --- | --- |
| <5 years | 2509 | 407 | 0.162 | 0.0016 | 0.0003 | 0.0019 | 0.142 |
| 5-15 years | 2509 | 764 | 0.304 | 0.0025 | 0.0007 | 0.0019 | 0.403 |
| ≥ 16 years | 2509 | 1338 | 0.533 | 0.002 | 0.0008 | 0.0019 | 0.455 |
