# Supplementary material for: Asymptomatic school children and adults are important for the human infectious reservoir for Plasmodium falciparum malaria in an area of low endemicity in The Gambia
Source: J Infect. 2025 Jul;91(1):106507. doi: 10.1016/j.jinf.2025.106507 (PMC12170349; doi:10.1016/j.jinf.2025.106507)
Supplement: Supplementary file 2 — Supplementary material [file mmc2.docx]

**Supplementary figure 1**. **Fitted lines for the association of gametocyte density with percentage of mosquitoes infected in The Gambia and Uganda.** The relationship between gametocyte density and the proportion of mosquitoes infected, dots represent the data points from the current study conducted in The Gambia. Fitted lines represent the best fitted association for current data from The Gambia (grey line) and previously published data from Uganda (green line), shaded areas represent the 95% CIs. Data from Uganda is from a cohort study in a low endemic setting in Eastern Uganda [14].

**Supplementary figure 2**. Contribution of different infection types (symptomatic vs. asymptomatic sub-microscopic and asymptomatic microscopic) in the infected population stratified by cut-off value of microscopy (<100 parasites/µL by 18S qPCR) in relation to fever in the last 24 hours (A) and last 7 days (B). The contribution of asymptomatic submicroscopic, asymptomatic microscopic and symptomatic infections to the infectious reservoir was determined based on measured mosquito infection rates if available and, if unavailable, by imputing mosquito infection rates for samples with known gametocyte densities. Bar heights represent the proportion of infected mosquitoes, bar widths the proportion of each infection types in the infected population. Sample size corresponding to bar widths is described in the appendix methods. The percentage indicated above each bar is the contribution of each infection type to the infectious reservoir.
