# Supplementary figures and images for: Asymptomatic school children and adults are important for the human infectious reservoir for Plasmodium falciparum malaria in an area of low endemicity in The Gambia
Source: J Infect. 2025 Jul;91(1):106507. doi: 10.1016/j.jinf.2025.106507 (PMC12170349; doi:10.1016/j.jinf.2025.106507)

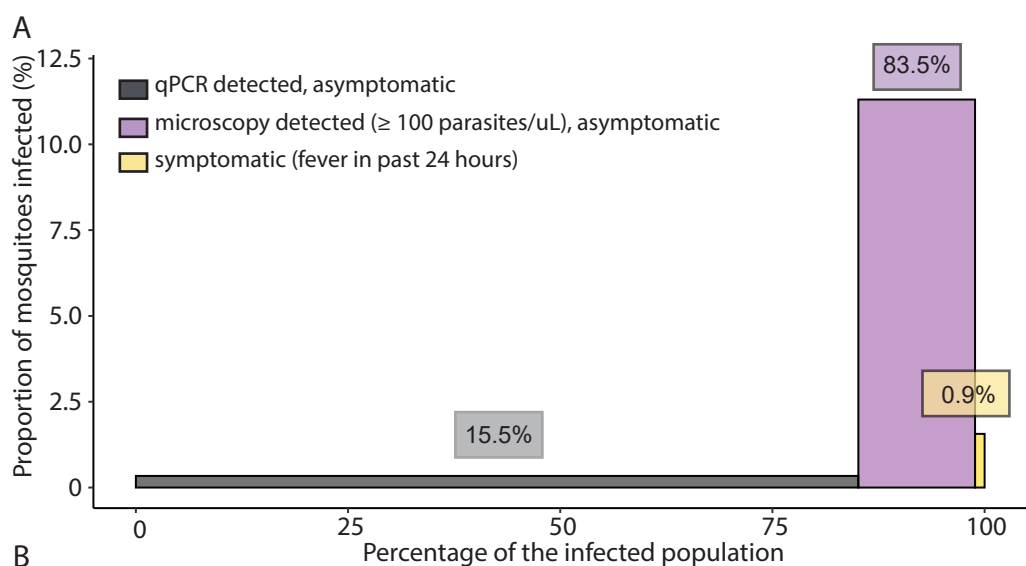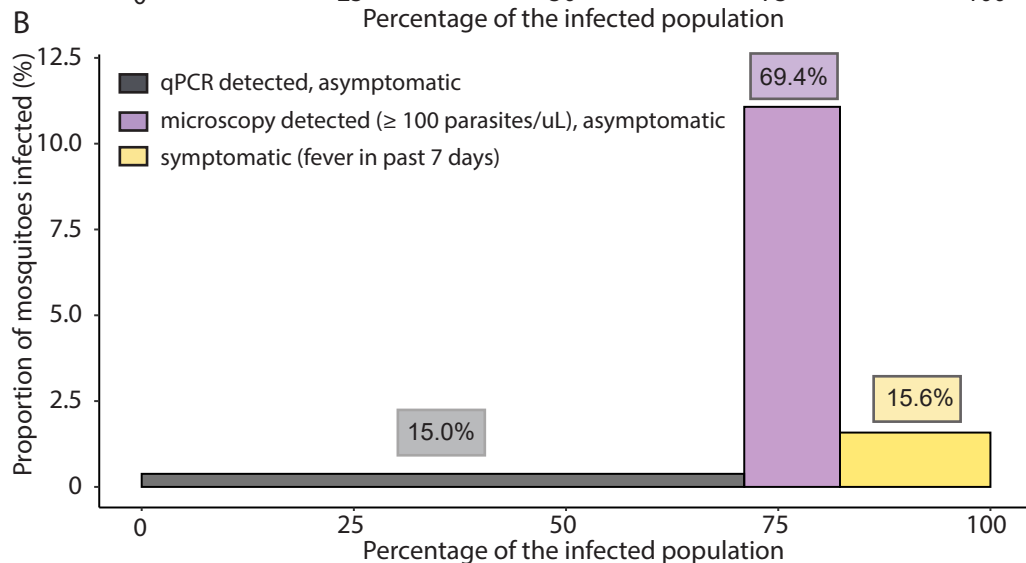

Supplement: Supplementary file 3 — Supplementary material [file mmc3.pdf]

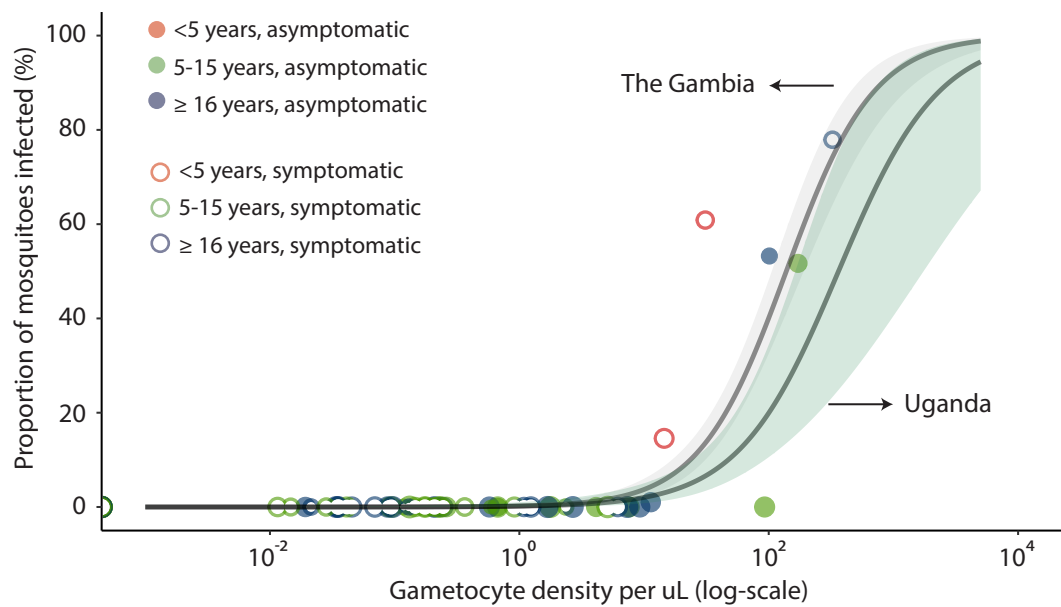

Supplement: Supplementary file 4 — Supplementary material [file mmc4.pdf]
